# Molecular characterization of *Dipetalonema yatesi* from the black-faced spider monkey (*Ateles chamek*) with phylogenetic inference of relationships among *Dipetalonema* of Neotropical primates

**DOI:** 10.1016/j.ijppaw.2022.01.005

**Published:** 2022-01-13

**Authors:** Daniel A. Zárate-Rendón, Michelle N. Salazar-Espinoza, Stefano Catalano, Caroline Sobotyk, Ana Patricia Mendoza, Marieke Rosenbaum, Guilherme Verocai

**Affiliations:** a Laboratorio de Parasitología, Departamento Académico de Nutrición, Facultad de Zootecnia, Universidad Nacional Agraria La Molina, Lima, 12, Peru; b Moredun Research Institute, Pentlands Science Park, Penicuik, EH26 0PZ, UK; c Department of Veterinary Pathobiology, College of Veterinary Medicine and Biomedical Sciences, Texas A&M University, College Station, TX, 77843, USA; d Department of Biology, University of Missouri – St. Louis, St. Louis, MO, 63121, USA; e Neotropical Primate Conservation, Moyobamba, San Martín, 22001, Peru; f Department of Infectious Disease and Global Health, Cummings School of Veterinary Medicine at Tufts University, North Grafton, MA, 01536, USA

**Keywords:** Dipetalonema, Filariosis, Filarioidea, Neotropics, Onchocercidae, Peruvian amazon, Phylogeny

## Abstract

Species of the genus *Dipetalonema* are parasitic nematodes of the family Onchocercidae (Nematoda; Filarioidea) which infect the peritoneal cavity of Neotropical primates. Of these, six species have been taxonomically described, two of these have been reported infecting the black-faced spider monkey (*Ateles chamek*): *Dipetalonema gracile* and *Dipetalonema yatesi*. Description of *Dipetalonema* species have been based on morphological characteristics, and their phylogenetic relationships remain unresolved. A few molecular studies have been carried out in *Dipetalonema* spp. infecting Neotropical primates. Seven filarioid nematodes (6 females and one male) recovered from one *A. chamek* in the Peruvian Amazon rainforest were morphologically identified as *D. yatesi* and molecularly characterized. A multi-locus genetic analysis of nuclear ribosomal region (18S) and mitochondrial (*cox1*, 12S, and *nad5*) gene sequences supported *D. yatesi* as a distinct lineage and yielded a highly resolved phylogenetic lineage tree for this filarioid genus of Neotropical primates. Our results highlighted that *Dipetalonema* species are divided in two well-supported clades, one containing *D. yatesi* and *D. caudispina*, and the second containing *D. robini*, *D. gracile,* and *D. graciliformis*. Due to sequence ambiguities from GenBank entries, relationships among isolates of *D. gracile* and *D. graciliformis* cannot be fully resolved, which requires further investigation. However, this suggests that these could represent a species complex. Our study confirms that *D. yatesi* is a valid species and constitutes the first molecular phylogenetic analysis of this parasite in black-faced spider monkeys.

## Highlights

• *Dipetalonema yatesi* from the black-faced spider monkey (*Ateles chamek*) in Peru.
• Characterization of *D. yatesi* nuclear ribosomal and mitochondrial gene sequences.
• Phylogenetic inference of relationships among *Dipetalonema* species.
• *Dipetalonema yatesi* and *D. caudispina* form a well-supported clade.
• Relationships among *D. gracile* and *D. graciliformis* isolates are not fully resolved.

## Graphical abstract

**Figure:**
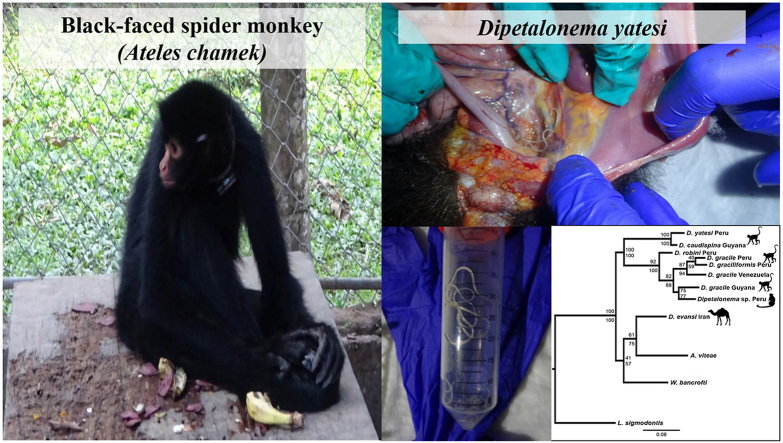

## 1 Introduction

Non-human primates constitute a diverse group of species living in tropical and sub-tropical regions of America, Africa, and Asia, with only few species adapted to temperate climates (Dolhinow and Fuentes, 1999). Due to their close relationship to human beings, they were subjected to numerous studies for their role as reservoir for pathogens, including parasitic nematodes (Cañizales and Guerrero, 2017; Solórzano-García and Pérez-Ponce de León, 2018). Primates are particularly vulnerable to the effects of parasitic infections since their tight social lifestyle (Freeland, 1983). The Atelidae comprises the largest family of monkeys across South and Central America (Rylands et al., 2012). Atelid monkeys are currently grouped into two subfamilies (i.e., Alouattinae and Atelinae) and five genera (i.e., *Alouatta*, *Ateles*, *Brachyteles*, *Lagothrix*, and *Oreonax*). The taxonomy within the genus *Ateles* has changed considerably (Morales-Jimenez et al., 2015). For instance, the black-faced spider monkey *Ateles chamek* was first described as *Ateles paniscus chamek* (see Morales-Jimenez et al., 2015) before it was recognized as a separate species (Wallace, 2008).

Filarioid nematodes are parasites that belong to the Superfamily Filarioidea (Order Spirurida) and infect tissues and body cavities of vertebrate hosts (Anderson et al., 2000). All filarioids have an indirect life-cycle, requiring an arthropod intermediate host for development and transmission. In addition to the recent molecular report of an unidentified *Brugia* species from the red howler monkey in French Guiana (Laidoudi et al., 2020), two other genera within the Family Onchocercidae, *Mansonella* and *Dipetalonema,* have been reported infecting nonhuman primates in the Americas (Bain et al., 1986; Laidoudi et al., 2020). Adult nematodes within the genus *Dipetalonema* parasitize the peritoneal cavity of their definitive hosts, while their microfilariae are found circulating in the bloodstream. Cavitary *Dipetalonema* infections can cause mild inflammatory reactions, including peritonitis and pleuritis with fibrinous adhesions (Strait et al., 2012). To date, biting midges of the genus *Culicoides* (Arthropoda: Ceratopogonidae) are the only biologically confirmed intermediate hosts and biological vectors of *Dipetalonema* (Eberhard et al., 1979; Travi et al., 1985; Notarnicola et al., 2007). There are six species in the genus *Dipetalonema* which parasitize Neotropical primates: *D. gracile* (Rudolphi, 1809); *D. caudispina* (Molin, 1858); *D. graciliformis* Freitas (1964); *D. robini* Petit, Bain, and Roussilhon, 1985; *D. freitasi* Bain et al. (1987); and *D. yatesi* Notarnicola et al. (2007) (Vanderhoeven et al., 2017). These nematodes have been isolated from over 20 species of monkeys from nine different genera of Neotropical primates of the tribe Platyrrhini. However, the true geographic distribution of many of these species is unknown since most reports of *Dipetalonema* infection in Neotropical primates come from animals in captivity (Conga et al., 2018). Only two *Dipetalonema* species have been reported in the black-faced spider monkey *A. chamek*: *D. gracile*, found in the Noel Kempff Mercado National Park, Bolivia (Karesh et al., 1998), and *D. yatesi*, a newly described species first isolated in north-eastern Bolivia (Notarnicola et al., 2007). The description of this latter species was solely based in morphological characteristics (i.e., structure and dimensions of the spicules and gubernaculum in males; morphology of the vulva and posterior end in females) (Notarnicola et al., 2007). As no molecular data are available for *D. yatesi*, much remains unclear regarding its phylogenetic relationships with other congeneric species.

Few molecular studies focusing on the genus *Dipetalonema* have been performed and most have been related to *D. gracile*. The present work integrated morphological and molecular analyses within a phylogenetic framework to confirm *D. yatesi* as a valid species infecting the peritoneal cavity of *A. chamek* in the Peruvian Amazon and highlighted new features on the evolutionary relationships among species of the genus *Dipetalonema*.

## 2 Materials and methods

### 2.1 Specimens collection

**Fig. 1:**
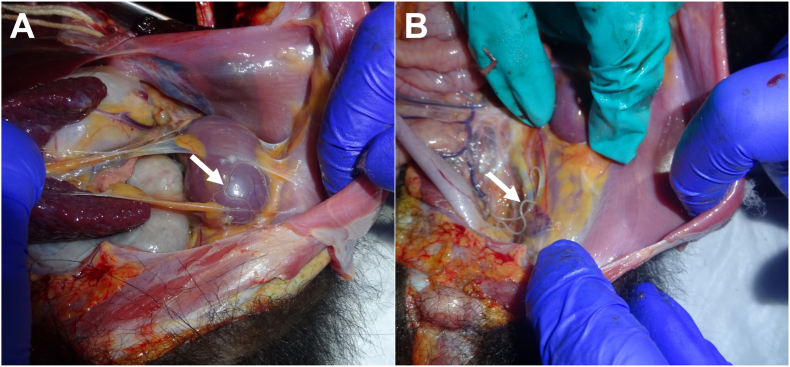
Macroscopic observation of *Dipetalonema yatesi* on the capsule of the left kidney (A) and on the parietal peritoneum (B) at the post-mortem examination of a black-faced spider monkey (*Ateles chamek*).

A total of seven specimens of filarioid nematodes were collected from the abdominal cavity of a juvenile male black-faced spider monkey at Taricaya Rescue Center in Madre de Dios, southern Peruvian Amazon (12° 31′ 09″ S, 68° 58′ 47″ W). This monkey was confiscated by Peruvian authorities from wildlife trafficking in Puerto Maldonado and sent to the program for the rehabilitation and reintroduction of spider monkeys at Taricaya Rescue Center. It arrived in good physical condition and was apparently healthy but was euthanized six months later due to a chronic herpesvirus infection detected during quarantine. Necropsy findings included multifocal pneumonia, pleural adhesions, ascites, and multiple cysts of approximately 5 mm of diameter with fibrinous adhesions in the mesenteries, peritoneum, and retroperitoneal spaces. A large number of filarioid nematodes of 10–15 cm in length were observed infecting the abdominal cavity (Fig. 1). A total of seven specimens (6 females and 1 male) were collected and stored in 96% ethanol for morphological and molecular identification as described below.

Specimens’ collection was authorized by the Peruvian government through the research permit RDG #067-2020-MINAGRI-SERFOR/DGGSPFFS and export permit CITES #21 PE003987/SP.

### 2.2 Laboratory analyses

For morphological identification, fragments of the anterior and posterior extremities of three specimens were cut using a sterile scalpel blade, cleared in lactophenol for 1 h, and subsequently mounted for observation under an Olympus BX53 optical microscope at 10X, 20X and 40× magnification.

One female and one male parasite were used for molecular analysis. Genomic DNA was extracted using the DNeasy® Blood & Tissue Kit (QIAGEN, Hilden, Germany) following the manufacturer's instructions. DNA extracts were amplified for the partial 18S region of the nuclear ribosomal DNA (rDNA), the partial mitochondrial 12S gene sequence of the ribosomal RNA (rRNA), the partial cytochrome *c* oxidase subunit 1 (*cox1*) and NADH dehydrogenase subunit 5 (*nad5*) of the mitochondrial DNA (mtDNA). Polymerase chain reaction (PCR) was performed in 25 μL reactions containing 0.25 μM of each primer, 1x GoTaq® Green Master Mix (Promega Corporation, Madison, WI, USA) and 2.5 μL of DNA template. We amplified the 18S rDNA using two primer pairs based on previously published sequences (Floyd et al., 2005; Lefoulon et al., 2015). The primers F18ScF1 (5′-ACCGCCCTAGTTCTGACCGTAAA-3′) and F18ScR1 (5′- CTCTGGCTTCATCCTGCTCA-3′) were used under the following cycling conditions: initial denaturation 95 °C for 2 min, followed by 40 cycles of 95 °C for 30 s, 58 °C for 45 s, and 72 °C for 90 s, and final extension at 72 °C for 5 min. The primer pair Nem_18S_F (5′- CGCGAATRGCTCATTACAACAGC-3′) and Nem_18S_R (5′- GGGCGGTATCTGATCGCC-3′) were used under the following cycling conditions: initial denaturation 95 °C for 2 min, followed by 35 cycles of 95 °C for 30 s, 54 °C for 30 s, and 72 °C for 1 min, and final extension at 72 °C for 5 min. The 12S rRNA region was amplified using primers 12SF (5′-GTT CCA GAA TAA TCG GCT A-3′) and 12SR (5′-ATT GAC GGA TGR TTT GTA CC-3′) (Casiraghi et al., 2004) under the following cycling conditions: initial denaturation 95 °C for 2 min, followed by 40 cycles of 95 °C for 40 s, 50 °C for 45 s, and 72 °C for 90 s, and final extension at 72 °C for 5 min. The *cox1* mtDNA gene sequence was amplified using COIintF (5′-TGA TTG GTG GTT TTG GTA A-3′) and COIintR (5′-ATA AGT ACG AGT ATC AAT ATC-3′) (Casiraghi et al., 2001) under the following cycling conditions: initial denaturation 95 °C for 2 min, followed by 40 cycles of 95 °C for 45 s, 52 °C for 45 s, and 72 °C for 90 s, and final extension at 72 °C for 5 min. The *nad5* mtDNA gene sequence was amplified using ND5-Ov5A-F (5′-TTG GTT GCC TAA GGCTAT GG-3′) and ND5OvC-R (5′-CCC CTA GTA AACAAC AAA CCA CA-3′) (Morales-Hojas et al., 2006) under the following cycling conditions: initial denaturation 95 °C for 2 min, followed by 40 cycles of 95 °C for 30 s, 50 °C for 45 s, and 72 °C for 45 s, and final extension at 72 °C for 5 min. PCR products were purified using E.Z.N.A.® Cycle Pure Kit (Omega Bio-tek, Norcross, GA, USA), then sequenced in both directions using the original PCR primers in a 3730xl DNA Analyzer at Eurofins Genomics (Louisville, KY, USA). We assembled and edited contigs using CodonCode Aligner v9.0.1 (CodonCode Corporation, Centerville, MA, USA). These data, together with previously published sequences available in the GenBank™ database (Table 1), were aligned using MUSCLE (Edgar, 2004) as implemented in CodonCode Aligner v9.0.1 since no internal gaps were present. The 12S data were aligned using ProAlign v0.5 (Löytynoja and Milinkovitch, 2003) and 60% minimum posterior probability of sites as the criterion for detecting and removing unreliably aligned characters.

**Table 1:** List of *Dipetalonema* spp. and outgroups used in our study, including sampling locality, host species, GenBank™ accession numbers and base pair (bp) length for the 18S of the nuclear ribosomal DNA, 12S of the ribosomal RNA, and cytochrome *c* oxidase subunit 1 (*cox1*) of the mitochondrial DNA (NA when not available).

Parasite	Country	Host	Accession 18S (bp)	Accession 12S (bp)	Accession *cox1* (bp)	Reference
*Dipetalonema*						
*D. caudispina*	Guyana	*Ateles paniscus*	KP760126 (614)	KP760323 (420)	KP760177 (632)	Lefoulon et al. (2015)
*D. caudispina*	Guyana	*Ateles* sp.	KP760127 (629)	KP760324 (422)	KP760178 (632)	Lefoulon et al. (2015)

*D. gracile*	Venezuela	*Cebus olivaceus*	KP760128 (665)	KP760325 (419)	KP760179 (628)	Lefoulon et al. (2015)
*D. gracile*	Peru	*Sapajus macrocephalus*	KP760129 (665)	KP760326 (422)	KP760180 (613)	Lefoulon et al. (2015)
*D. gracile*	Guyana	*Ateles* sp.	KP760130 (648)	KP760327 (423)	KP760181 (586)	Lefoulon et al. (2015)
*D. graciliformis*	Peru	*Saimiri sciureus*	KP760131 (665)	KP760328 (421)	KP760182 (632)	Lefoulon et al. (2015)
*D. robini*	Peru	*Lagothrix poeppigii*	KP760132 (665)	KP760329 (390)	KP760183 (632)	Lefoulon et al. (2015)
*D. yatesi*	Peru	*Ateles chamek*	MW192232-3 (1,618)	MW209693-4 (398)	MW199182-3 (649)	Present study
*Dipetalonema* sp.	Peru	*Leontocebus fuscicollis*	NA	NA	KX932481 (626)	Erkenswick et al. (2017)
*Dipetalonema* sp.	Peru	*Saguinus imperator*	NA	NA	KX932482 (627)	Erkenswick et al. (2017)
*Deraiophoronema*						
*D. evansi*	Iran	*Camelus dromedarius*	NA	NA	KR184801-18 (674)	Sazmand et al. (2016)

Alignments of the 18S, 12S, and *cox1* were analysed both separately and as concatenated data. We concatenated the sequences, and partitioned the datasets, using SequenceMatrix v1.8 (Vaidya et al., 2011) after executing an incongruence length difference (ILD) test (Farris et al., 1995) in PAUP* v4.0a (Sinauer Associates, Sunderland, MA, USA) to assess homogeneity between partitions. We performed the ILD test using 100,000 replicates, random addition of sequences (10 replicates), and the tree-bisection-reconnection algorithm for branch swapping. We inferred phylogenetic relationships by executing maximum likelihood (ML) in RAxML v8.2 (Stamatakis, 2014) and Bayesian inference (BI) in MrBayes v3.2.6 (Ronquist et al., 2012) as implemented in the Cyberinfrastructure for Phylogenetic Research (CIPRES) web portal (http://www.phylo.org). We used PartitionFinder v2.1.1 (Lanfear et al., 2016) to select the best-fit evolutionary models. For the ML analysis we enforced a generalized time-reversible (GTR) substitution model with rate heterogeneity across all partitions (i.e., 18S, 12S, and *cox1*'s first, second, and third codon positions), selected automatic arrest of bootstrap resampling to assess nodal support, and specified outgroups belonging to the family Onchocercidae (i.e., *Acanthocheilonema vietae* (GenBank™ accession numbers DQ094171 and HQ186249), *Litomosoides sigmodontis* (GenBank™ accession numbers AP227233 and AP017689), and *Wuchereria bancrofti* (GenBank™ accession numbers AY843436 and JQ316200)). For the BI analysis we enforced a Kimura Two-Parameter (K2P) substitution model with rate heterogeneity across the 18S, a GTR model with invariable sites across the 12S, a Hasegawa-Kishino-Yano (HKY) model with invariable sites across the *cox1*'s first and third codon positions, and a HKY model with rate heterogeneity across the *cox1*'s second codon position. The BI analysis was performed without specifying any outgroup and using two independent runs with four Markov Chain Monte Carlo (MCMC) chains and 10 million generations. MCMC chains were sampled every 10,000 generations and the first 25% of the trees was discarded as burn-in. The trees remaining after burn-in were used to create a 50% majority-rule consensus tree with posterior probabilities indicating nodal support. The resulting tree topologies were visualized using FigTree v1.4.3 (http://tree.bio.ed.ac.uk/software/figtree/).

## 3 Results

**Fig. 2:**
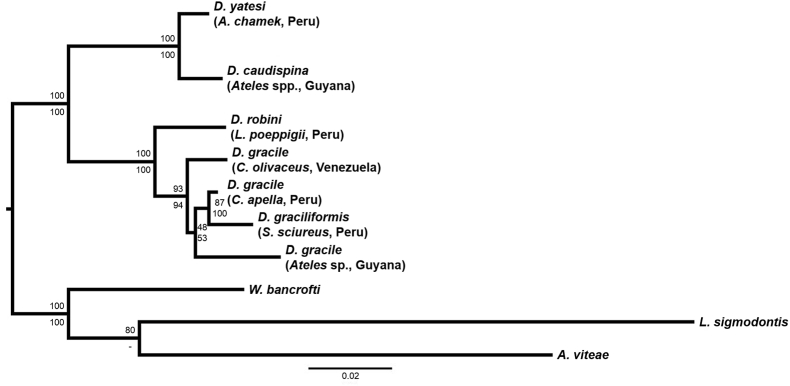
Phylogenetic relationships among species of *Dipetalonema* spp. infecting non-human primates (i.e., *Ateles* spp., *Cebus* spp., *Lagothrix poeppigii*, and *Saimiri sciureus*) using a concatenated dataset of 1615 base pairs including the 18S of the nuclear ribosomal DNA, 12S of the ribosomal RNA, and cytochrome *c* oxidase subunit 1 (*cox1*) of the mitochondrial DNA. The taxa *Acanthocheilonema viteae*, *Litomosoides sigmodontis*, and *Wuchereria bancrofti* were used as outgroups. At each branch, the nodal support is represented by the maximum likelihood percentage above and the Bayesian posterior probability below (the hyphen indicates when support is missing).

**Fig. 3:**
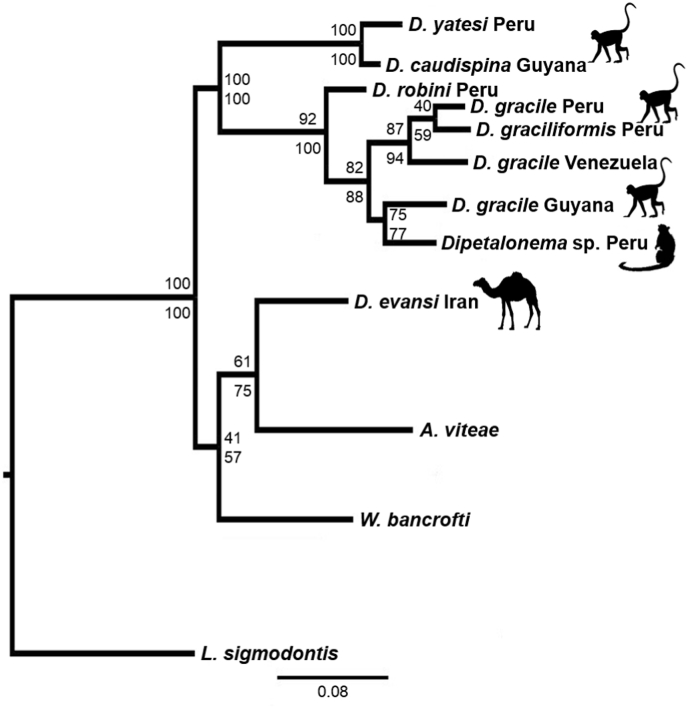
Phylogenetic relationships among species of *Dipetalonema* using a dataset of 586 base pairs including the partial cytochrome *c* oxidase subunit 1 (*cox1*) of the mitochondrial DNA. The black silhouettes of the monkey, tamarin, and camelid indicate the hosts from which the filarioid nematodes were isolated. The taxa *Acanthocheilonema viteae*, *Litomosoides sigmodontis*, and *Wuchereria bancrofti* were used as outgroups. At each branch, the nodal support is represented by the maximum likelihood percentage above and the Bayesian posterior probability below.

The nematode specimens were identified as *D. yatesi* under microscopy based on published identification keys (Notarnicola et al., 2007). Specimens were deposited in the Museo de Historia Natural de la Universidad Nacional Federico Villarreal, Lima, Peru (Accession number: MUFV: ZOO-HPIA 205). Two specimens of *D. yatesi* were sequenced for their 18S rDNA (1642 base pairs (bp)), 12S rRNA (398 bp), *cox1* mtDNA (649 bp), and *nad5* mtDNA (428 bp). The 12S alignment excluded 33 of 405 sites based on posterior probability filtering. The ILD test validated the concatenation of the partitions since the null hypothesis of congruence was rejected (*P* = 0.36). Phylogenetic analysis of the concatenated alignment (1615 bp), which included 18S, 12S, and *cox1* datasets (657 bp, 372 bp, and 586 bp, respectively), yielded a single best-scoring tree strongly supporting *D. yatesi* as a distinct lineage. ML bootstrap values and BI posterior probabilities supported a highly resolved topology indicating two clades, one of which was composed by parasites of spider monkeys (i.e., *D. caudispina* and *D. yatesi*) (Fig. 2). The second clade contains *D. robini*, *D. gracile,* and *D. graciliformis*. Relationships among isolates within the *D. gracile*/*D. graciliformis* clade are not fully resolved. Both ML and BI analyses of each aligned dataset (i.e., 18S rDNA, 12S rRNA, and *cox1* mtDNA) yielded similar, although less defined, relationships among *Dipetalonema* species. Nevertheless, the phylogenetic tree for the *cox1* dataset confirmed the revised identification of *Dipetalonema evansi* as *Deraiophoronema evansi* (Sazmand et al., 2016, 2019; Bilegjargal et al., 2021), a filarioid nematode that infects Old World camelids, since this taxon failed to cluster within the *Dipetalonema* complex of species (Fig. 3).

We deposited molecular sequences of *D. yatesi* in GenBank™ under the accession numbers MW192232 and MW192233 (18S rDNA), MW209693 and MW209694 (12S rRNA), MW199182 and MW199183 (*cox1* mtDNA), and MW194891 and MW194892 (*nad5* mtDNA).

## 4 Discussion

Our work provides the first report of *D. yatesi* in *A. chamek* from Peru after its original description, which was based only on morphology and morphometry of specimens isolated from the same host in Bolivia (Notarnicola et al., 2007). The geographic distribution of the black-faced spider monkey ranges from north-eastern Peru and north-central Bolivia to areas of the Brazilian Amazonian rainforest in the states of Acre, Rondônia, Mato Grosso, and Amazonas (Rabelo et al., 2014, 2018) To date, there have been no reports of *D. yatesi* in *A. chamek* from Brazil and other areas of Peru and Bolivia, but this might be due to a lack of surveillance associated with the somewhat recent description of the species. In contrast, *D. gracile* was reported in *A. chamek* from Bolivia (Karesh et al., 1998) and Peru (Dunn and Lambrecht, 1963), but these specimens were identified only based on morphology. Current knowledge suggests that *D. yatesi* may be host specific, whereas *D. gracile* has been reported not only in *A. chamek* but also other *Ateles* species, including *A. paniscus* from Brazil, Panama, and Peru (Caballero, 1947; Freitas, 1964), *Ateles geoffroyi* from Mexico and Panama (Caballero, 1947; Lamothe-Argumedo et al., 1997), *Ateles fusciceps* from Panama (McCoy, 1936; Caballero, 1947), and *Ateles nigricollis* from Panama (Dunn and Lambrecht, 1963). The relatively recent advances in the knowledge of the biodiversity and taxonomy of *Dipetalonema* of Neotropical primates are aiding species-specific diagnostics and are providing insight into host-parasite co-evolutionary history (Lefoulon et al., 2015, 2017; Milstein et al., 2020; Zhang et al., 2020).

Currently, there are six valid species in the *Dipetalonema* genus, with the addition of *D. robini*, *D. freitasi*, and *D. yatesi*. Our analysis supports *D. yatesi* as the sister species of *D. caudispina*, indicating that filarioid nematodes of spider monkeys form a well-supported clade. Currently, there are no genetic data available for *D. freitasi* and therefore its phylogenetic relationships with other species within the genus cannot be inferred molecularly. However, the morphological similarity between *D. freitasi*, *D. yatesi*, and *D. caudispina*, as females belonging to these three species possess a sinuous vagina vera (Notarnicola et al., 2007), suggest a close phylogenetic relationship among them.

Many early reports of *Dipetalonema* in Neotropical primates were assumed to belong to *D. caudispina* and *D. gracile* since these were the only known species for decades. Therefore, historical records may have biased the rather broad host range of these filarioid species. For instance, *D. caudispina* has been reported in nine primate species belonging to nine genera across three families (i.e., Atelidae, Callithricidae, and Cebidae). Similarly, *D. gracile* has been reported in at least 16 species belonging to seven genera within four families (i.e., Atelidae, Aotidae, Cebidae, and Pitheciidae) (Notarnicola et al., 2008; Conga et al., 2018, 2019a). While it is possible that both species are host generalists, and even have been found in co-infections (Conga et al., 2019b), our phylogenetic analysis suggests that *D. gracile/D. graciliformis* may represent a species complex. The type-host for *D. gracile* is the capuchin *Cebus capucinus* (Cebidae) and for *D. graciliformis* the tamarin *Saguinus midas* (Callithricidae) (Freitas, 1943, 1964; Bain et al., 1987). Recent molecular studies based on partial cox1 sequences support *D. graciliformis* as a multi-host species, infecting *S. midas* and *Saimiri sciureus* (Cebidae) (Lefoulon et al., 2015; Milstein et al., 2020; Laidoudi et al., 2021).

All currently available sequences of *D. caudispina* come from specimens isolated from its type-host, *A. paniscus* (Lefoulon et al., 2015; Milstein et al., 2020), however the original description of *D. caudispina* by Molin listed various primate species as potential hosts (Freitas, 1943). The material used by Freitas (1943) to confirm *D. caudispina* as a valid species originated only from *A. paniscus*. Therefore, future molecular characterizations of putative *D. caudispina* from different hosts may also reveal cryptic diversity. In summary, the current knowledge on the host-parasite associations of *D. caudispina*, *D. gracile*, and *D. graciliformis* should be interpreted cautiously and should be revisited through integrated classical and molecular methods, ideally including material from the type hosts and type localities of each filarioid species.

Further investigations based on integrated morphological and molecular approaches are required to shed further light into the diversity, host associations, and geographic distribution of *Dipetalonema* species infecting Neotropical primates. Nevertheless, there are numerous challenges for robust and comprehensive sampling of adult nematode specimens through necropsy, including the remote locations and the conservation status of many of the host species. A potential strategy to overcome some of these knowledge gaps is the application of less invasive and non-terminal methods for sample collection. Screening and characterizing microfilariae found in blood of animals that are captured or rescued could assist in answering some of the abovementioned questions (Laidoudi et al., 2021). Infections by filarioid nematodes in South American non-human primates is well known to local and indigenous communities despite the scant scientific reports of *D. yatesi* and other *Dipetalonema* species (Milstein et al., 2020). Furthermore, microfilariae are commonly found in blood smears of rescued *A. chamek* (P. Mendoza, pers. comm.) and several species of confiscated primates in Peru (Zariquiey Morcos, 2014). Filarioid infections have been described as benign in Neotropical primates (Chalifoux, 1993); however, the intensity of *Dipetalonema* spp. infections, observed at the post-mortem examination of animals clearly showing clinical symptoms (e.g., our current study; Karesh et al., 1998; Milstein et al., 2020), suggests that these parasitic infections may contribute as a co-morbidity factor in captive settings (such as rescue centres, wet markets, and confiscation facilities) in which vectors are abundant and primate ecology has been severely disrupted (Shanee et al., 2017).

Other aspects of the biology of *D. yatesi* remain unknown (Notarnicola et al., 2007, 2008). While *Culicoides* biting midges have been biologically proven to serve as intermediate hosts for other *Dipetalonema* species infecting Neotropical primates, there have been no studies assessing their role in the cycle of *D. yatesi* (Eberhard et al., 1979; Travi et al., 1985). Nevertheless, the molecular markers characterized in the present study may be useful for the xenomonitoring of *Culicoides* and other potential dipteran vectors, including their application to broader studies on the epidemiology of filarioid parasites.

## 5 Conclusions

Integrating the rapid collection of molecular data with opportunistic sampling is a vital effort to further non-invasive disease diagnostics and ecological knowledge of *A. chamek* and other endangered wildlife populations which continue to decline, primarily due to deforestation and hunting pressure (Solórzano-García and Pérez-Ponce de León, 2018; Bogoni et al., 2020). Our study expands the known range of *D. yatesi,* which was previously only recorded in the black-faced spider monkey *A. chamek* in northern Bolivia, to southern Peru. Our phylogenetic analysis confirms that *D. yatesi* is a valid species that is closely related to *D. caudispina*, resolves phylogenetic relationships among within *Dipetalonema* species, and highlights the potential for hidden diversity within the genus.

## Declaration of competing interest

The authors declare no conflict of interest.

## References

[bib1] Anderson R., Chabaud A., Willmot S. (2000).

[bib2] Bain O., Petit G., Rosales-Loesener L. (1986). Filaires de singes sud-américains. Bull. Mus. Natl. Hist. Nat., Paris section A.

[bib3] Bain O., Diagne M., Muller R. (1987). Une cinquième Filaire du genre *Dipetalonema*, parasite de singes sud-américains. Ann. Parasitol. Hum. Comp..

[bib4] Bilegjargal J., Rzad I., Fukumoto S., Chinchuluun B., Lkhagvatseren S., Gantuya S., Azjargal G., Batsukh Z., Munkhjargal T. (2021). Microscopic and molecular detection of *Deraiophoronema evansi* (Lewis, 1882) in domestic Bactrian camels (*Camelus bactrianus*) of Mongolia. Parasitol. Int..

[bib5] Bogoni J.A., Peres C.A., Ferraz K.M.P.M.B. (2020). Extent, intensity and drivers of mammal defaunation: a continental-scale analysis across the Neotropics. Sci. Rep..

[bib6] Caballero Y.C.E. (1947).

[bib7] Cañizales I., Guerrero R. (2017). Artrópodos, protozoos, y helmintos parásitos de mamíferos silvestres (Mammalia) de Venezuela. Neotrop. Primates.

[bib8] Casiraghi M., Anderson T.J., Bandi C., Bazzocchi C., Genchi C. (2001). A phylogenetic analysis of filarial nematodes: comparison with the phylogeny of *Wolbachia* endosymbionts. Parasitology.

[bib9] Casiraghi M., Bain O., Guerrero R., Martin C., Pocacqua V., Gardner S.L., Franceschi A., Bandi C. (2004). Mapping the presence of *Wolbachia pipientis* on the phylogeny of filarial nematodes: evidence for symbiont loss during evolution. Int. J. Parasitol..

[bib10] Chalifoux L.V., Jones T.C., Mohr U., Hunt R.D. (1993). Nonhuman Primates I.

[bib11] Conga D.F., Mayor P., Furtado A.P., Giese E.G., dos Santos J.N. (2018). Occurrence of *Dipetalonema gracile* in a wild population of woolly monkey *Lagothrix poeppiigii* in the northeastern Peruvian Amazon. Rev. Bras. Parasitol. Vet..

[bib12] Conga D.F., Mayor P., Furtado A.P., Giese E.G., dos Santos J.N. (2019). First report of filarial nematodes in free-living pitheciid primates. Syst. Parasitol..

[bib13] Conga D.F., Mayor P., Furtado A.P., Giese E.G., dos Santos J.N. (2019). Co-infection with filarial nematodes in *Sapajus macrocephalus* and *Cebus albifrons* (primates: Cebidae) from the Peruvian Amazon. J. Helminthol..

[bib14] Dolhinow P., Fuentes A. (1999).

[bib15] Dunn F.L., Lambrecht F.L. (1963). On some filarial parasites of South American primates, with a description of *Tetrapetalonema tamarinae* n. sp. from the Peruvian tamarin marmoset, *Tamarinus nigricollis* (Spix, 1823). J. Helminthol..

[bib16] Eberhard M.L., Lowrie R.C., Orihel T.C. (1979). Development of *Dipetalonema gracile* and *D. caudispina* to the infective stage in *Culicoides hollensis*. J. Parasitol..

[bib17] Edgar R.C. (2004). MUSCLE: multiple sequence alignment with high accuracy and high throughput. Nucleic Acids Res..

[bib18] Erkenswick G.A., Watsa M., Gozalo A.S., Dmytryk N., Parker P.G. (2017). Temporal and demographic blood parasite dynamics in two free-ranging neotropical primates. Int. J. Parasitol.: Parasites Wildl..

[bib19] Farris J.S., Kallersjo M., Kluge A.G., Bult C. (1995). Constructing a significance test for incongruence. Syst. Biol..

[bib20] Floyd R.M., Rogers A.D., Lambshead P.J.D., Smith C.R. (2005). Nematode‐specific PCR primers for the 18S small subunit rRNA gene. Mol. Ecol. Notes.

[bib21] Freeland W.J. (1983). Parasited and the coexistence of animal host species. Am. Nat..

[bib22] Freitas J.F.T. (1943).

[bib23] Freitas J.F.T. (1964).

[bib24] Karesh W.B., Wallace R.B., Painter R.L.E., Rumiz D., Braselton W.E., Dierenfeld E.S., Puche H. (1998). Immobilization and health assessment of free-ranging black spider monkeys (*Ateles paniscus chamek*). Am. J. Primatol..

[bib25] Laidoudi Y., Medkour H., Levasseur A., Davoust B., Mediannikov O. (2020). New molecular data on filaria and its *Wolbachia* from red howler monkeys (*Alouatta macconnelli*) in French Guiana – a preliminary study. Pathogens.

[bib26] Laidoudi Y., Lia R.P., Mendoza-Roldan J.A., Modrý D., de Broucker C.A., Mediannikov O., Davoust B., Otranto D. (2021). *Dipetalonema graciliformis* (Freitas, 1964) from the red-handed tamarins (*Saguinus midas*, Linnaeus, 1758) in French Guiana. Parasitology.

[bib27] Lamothe-Argumedo R., García-Prieto L., Osorio-Sarabia D., Pérez-Ponce de León G. (1997).

[bib28] Lanfear R., Frandsen P.B., Wright A.M., Senfeld T., Calcott B. (2016). PartitionFinder 2: new methods for selecting partitioned models of evolution for molecular and morphological phylogenetic analyses. Mol. Biol. Evol..

[bib29] Lefoulon E., Bain O., Bourret J., Junker K., Guerrero R., Cañizales I., Kuzmin Y., Satoto T.B.T., Cardenas-Callirgos J.M., de Souza Lima S., Raccurt C., Mutafchiev Y., Gavotte L., Martin C. (2015). Shaking the tree: multi-locus sequence typing usurps current onchocercid (filarial nematode) phylogeny. PLoS Neglected Trop. Dis..

[bib30] Lefoulon E., Giannelli A., Makepeace B.L., Mutafchiev Y., Townson S., Uni S., Verocai G.G., Otranto D., Martin C. (2017). Whence river blindness? The domestication of mammals and host-parasite co-evolution in the nematode genus *Onchocerca*. Int. J. Parasitol..

[bib31] Löytynoja A., Milinkovitch M.C. (2003). A hidden Markov model for progressive multiple alignment. Bioinformatics.

[bib32] McCoy O.R. (1936). Filarial parasites of the monkeys of Panama. Am. J. Trop. Med..

[bib33] Milstein M.S., Shaffer C.A., Lindsey L.L., Wolf T.M., Suse P., Marawanaru E., Kipp E.J., Garwood T., Travis D.A., Terio K.A., Larsen P.A. (2020).

[bib34] Morales-Hojas R., Cheke R.A., Post R.J. (2006). Molecular systematics of five *Onchocerca* species (Nematoda: Filarioidea) including the human parasite, *O. volvulus*, suggest sympatric speciation. J. Helminthol..

[bib35] Morales-Jimenez A.L., Disotell T., Di Fiore A. (2015). Revisiting the phylogenetic relationships, biogeography, and taxonomy of spider monkeys (genus *Ateles*) in light of new molecular data. Mol. Phylogenet. Evol..

[bib36] Notarnicola J., Jiménez F.A., Gardner S.L. (2007). A new species of *Dipetalonema* (Filarioidea: Onchocercidae) from *Ateles chamek* from the Beni of Bolivia. J. Parasitol..

[bib37] Notarnicola J., Pinto C.M., Navone G.T. (2008). Host occurrence and geographical distribution of *Dipetalonema* spp. (Nematoda: Onchocercidae) in Neotropical monkeys and the first record of *Dipetalonema gracile* in Ecuador. Comp. Parasitol..

[bib38] Rabelo R.M., Silva F.E., Vieira T., Ferreira-Ferreira J., Paim F.P., Dutra W., Silva Júnior J.S., Valsecchi J. (2014). Extension of the geographic range of *Ateles chamek* (Primates, Atelidae): evidence of river-barrier crossing by an amazonian primate. Primates.

[bib39] Rabelo R., Gonçalves J., Silva F., Rocha D., Canale G., Bernardo C., Boubli J. (2018). Predicted distribution and habitat loss for the endangered black-faced black spider monkey *Ateles chamek* in the Amazon. Oryx.

[bib40] Ronquist F., Teslenko M., van der Mark P., Ayres D.L., Darling A., Höhna S., Larget B., Liu L., Suchard M.A., Huelsenbeck J.P. (2012). MrBayes 3.2: efficient Bayesian phylogenetic inference and model choice across a large model space. Syst. Biol..

[bib41] Rylands A.B., Mittermeier R.A., Alfaro J.W.L. (2012). Neotropical primates: taxonomy and recently described species and subspecies. Int. Zoo Yearbk..

[bib42] Sazmand A., Eigner B., Mirzaei M., Hekmatimoghaddam S., Harl J., Duscher G.G., Fuehrer H.P., Joachim A. (2016). Molecular identification and phylogenetic analysis of *Dipetalonema evansi* (Lewis, 1882) in camels (*Camelus dromedarius*) of Iran. Parasitol. Res..

[bib43] Sazmand A., Joachim A., Otranto D. (2019). Zoonotic parasites of dromedary camels: so important, so ignored. Parasites Vectors.

[bib44] Shanee N.A., Mendoza A.P., Shanee S. (2017). Diagnostic overview of the illegal trade in primates and law enforcement in Peru. Am. J. Primatol..

[bib45] Solórzano-García B., Pérez-Ponce de León G. (2018). Parasites of Neotropical primates: a review. Int. J. Primatol..

[bib46] Stamatakis A. (2014). RAxML version 8: a tool for phylogenetic analysis and post-analysis of large phylogenies. Bioinformatics.

[bib47] Strait K., Else J.G., Eberhard M.L., Abee C.R., Mansfield K., Tardif S., Morris T. (2012).

[bib48] Travi B.L., Eberhard M.L., Lowrie R.C. (1985). Development of *Dipetalonema gracile* in the squirrel monkey (*Saimiri sciureus*), with notes on its biology. J. Parasitol..

[bib49] Vaidya G., Lohman D.J., Meier R. (2011). SequenceMatrix: concatenation software for the fast assembly of multi‐gene datasets with character set and codon information. Cladistics.

[bib50] Vanderhoeven E., Notarnicola J., Agostini I. (2017). First record of *Dipetalonema robini* Petit, Bain & Roussilhon 1985 (Nematoda: Onchocercidae) parasitizing *Sapajus nigritus* in northeastern Argentina. Mastozool. Neotrop..

[bib51] Wallace R., Campbell C. (2008). Spider Monkeys: Behavior, Ecology and Evolution of the Genus *Ateles*.

[bib52] Zariquiey Morcos C.M. (2014).

[bib53] Zhang P., Ran R.K., Abdullahi A.Y., Shi X.L., Huang Y., Sun Y.X., Liu Y.Q., Yan X.X., Hang J.X., Fu Y.Q., Wang M.W., Chen W., Li G.Q. (2020). The mitochondrial genome of *Dipetalonema gracile* from a squirrel monkey in China. J. Helminthol..

